# mJustice: Preliminary Development of a Mobile App for Medical-Forensic Documentation of Sexual Violence in Low-Resource Environments and Conflict Zones

**DOI:** 10.9745/GHSP-D-16-00233

**Published:** 2017-03-24

**Authors:** Ranit Mishori, Michael Anastario, Karen Naimer, Sucharita Varanasi, Hope Ferdowsian, Dori Abel, Kevin Chugh

**Affiliations:** a Georgetown University School of Medicine, Washington, DC, USA.; b University of Central America, San Salvador, El Salvador.; c Physicians for Human Rights, Program on Sexual Violence in Conflict Zones, Boston, MA, USA.; d Hinckley Allen & Snyder LLP, Boston, MA, USA.; e Main Street Computing, Buffalo, NY, USA.

## Abstract

Digital health development and use has been expansive and operationalized in a variety of settings and modalities around the world, including in low- and middle-income countries. Mobile applications have been developed for a variety of health professionals and frontline health workers including physicians, midwives, nurses, and community health workers. However, there are no published studies on the development and use of digital health related to human rights fieldwork and to our knowledge no mobile health platforms exist specifically for use by frontline health workers to forensically and clinically document sexual violence. We describe a participatory development and user design process with Congolese end-users of a novel human rights app for clinicians intended to standardize the documentation of sexual violence evidence for forensic and legal purposes, called MediCapt. The app, yet to be launched and still in the future proofing phase, has included several development phases: (1) initial needs assessment conducted in 2011, (2) prototype development and field-testing in 2014 with 8 Congolese physicians, (3) prototype refinement and field-testing in 2015 with 9 clinicians. Feedback from the first field-testing phase was incorporated into the design of the second prototype; key features that were added to MediCapt include the ability for users to take photographs and draw on a pictogram to include as part of the evidence package, as well as the ability to print a form with the completed data. Questionnaires and key-informant interviews during the second and third field-testing phases revealed overall positive attitudes about MediCapt, but multiple perceived and actual barriers to implementation were identified, from personal behaviors, such as individual clinicians' comfort with new technology, to more systemic and infrastructure factors, such as strong cultural preferences for print documentation of evidence and limited Internet connectivity. Next phases of development include consideration of patients' acceptance of this technology, how it actually fits in the clinical workflow, and testing of how to transfer the collected evidence to law enforcement and legal authorities. Ultimately, we plan on conducting a robust evaluation to assess effectiveness of the app on medical, legal, and human rights outcomes. We believe our experience of collecting data that will potentially serve as legal evidence broadens the traditional scope of digital health and crosses a wide range of fields including medical, technological, legal, and ethical, and thus propose refining and defining this unique field of exploration as mobile justice, or mJustice.

## Abstract (precis)

The MediCapt mobile app has promise for clinicians to capture medical and forensic evidence of sexual violence and securely transmit the data to legal authorities for potential use in prosecution. We believe this application broadens the traditional scope of mHealth to collecting evidence, and thus name it mJustice.

## INTRODUCTION

The move toward a technology-centered world has changed the way we practice medicine and public health. This is manifested by the rapid expansion in the field of digital health, which concerns the "use of information and communications technologies to improve human health, healthcare services, and wellness for individuals and across populations."^1^ Within this broad field is the mobile health (mHealth) component that specifically uses mobile technologies such as mobile phones and personal digital assistants (PDAs).^2^

Between 2002 and 2016, an increasing number of digital health interventions were implemented around the world, many of them in developing countries.^3^ These projects have been developed for health professionals including frontline health workers (FHWs) such as physicians, midwives, nurses, and community health workers.^4^^–^^6^ A recent review found that digital health use enhances various aspects of FHWs' work activities and, in particular, that mobile phones help with data collection, reporting, communication, and health care delivery.^4^ However, most digital health projects in low- and middle-income countries are conducted at relatively small scale, in the form of pilot projects, and the evidence for a positive impact on health outcomes of many such pilots is mixed.^2^^,^^7^^–^^10^

Although digital health has experienced rapid expansion in recent years, there are no published studies on the development and use of digital health for human rights fieldwork. The gray literature reveals several existing smartphone and tablet applications (apps) related to human rights, but the majority provide educational and news materials^11^ and are not used for clinical or forensic data collection purposes.

Most existing mobile apps in the human rights field focus on providing educational and news content.

Extensive conversations with leaders in the field suggest there are 3 categories of technology tools being used in human rights communities: (1) those created to assist activists to gather information and documentation to expose or highlight violations, such as WITNESS' Video as Evidence program^12^ and Benetech's Martus tool^13^; (2) those aimed at securing or enhancing communication among activists and even providing protection, for example, Amnesty International's Panic Button app^14^; and (3) those directed at collecting, analyzing, and preserving evidence of crimes that could be admissible in a court, such as the International Bar Association's eyeWitness to Atrocities app.^15^ To our knowledge, no mobile health platforms have been developed specifically for use among FHWs to forensically and clinically document human rights violations, specifically sexual violence. Since these data would potentially serve as evidence admissible in courts, we consider this a new niche in the digital health field of data collection—one we propose terming mobile justice, or mJustice.

We propose defining the use of digital health technology to collect human rights-related evidence that could potentially be admissible in courts as mJustice.

In 2011, Physicians for Human Rights (PHR)—a U.S.-based NGO—launched the Program on Sexual Violence in Conflict Zones. One of the main activities of the program involved the development of a multisectoral training program for health, legal, and law enforcement professionals intended to enhance the forensic documentation and reporting of sexual violence incidents in the Democratic Republic of the Congo (DRC) and Kenya, among other places. Another major activity of the program has been to advocate the standardization of sexual violence medical information collection.

To that end, PHR, in conjunction with its local medical, law enforcement, and legal partners, has developed a Standard Sexual Violence form that is being used in parts of eastern DRC. The paper-based medical form was developed in-country with input from a network of doctors, lawyers, judges, and law enforcement officials, along with visiting international human rights scholars, practitioners, and experts. The information captured in the standard medical form is particular to the DRC and conforms to the local rule of law. The guiding principle behind the development of the form is that if the standard medical form is properly completed by a health professional trained in forensic medical evidence collection, more evaluations will be admissible in court, and consequently the success rate of prosecutions of sexual violence crimes will increase.

One of the major problems with the paper form of the standard medical certificate is the lack of proper storage, preservation, and ability to transfer it securely to the police and justice sector. Digitizing the standard medical form minimizes the chances of loss or theft of medical evidence, while preserving chain of custody. With these considerations in mind, we aimed to develop a smartphone-based app, called MediCapt.

**FIGURE 1:**
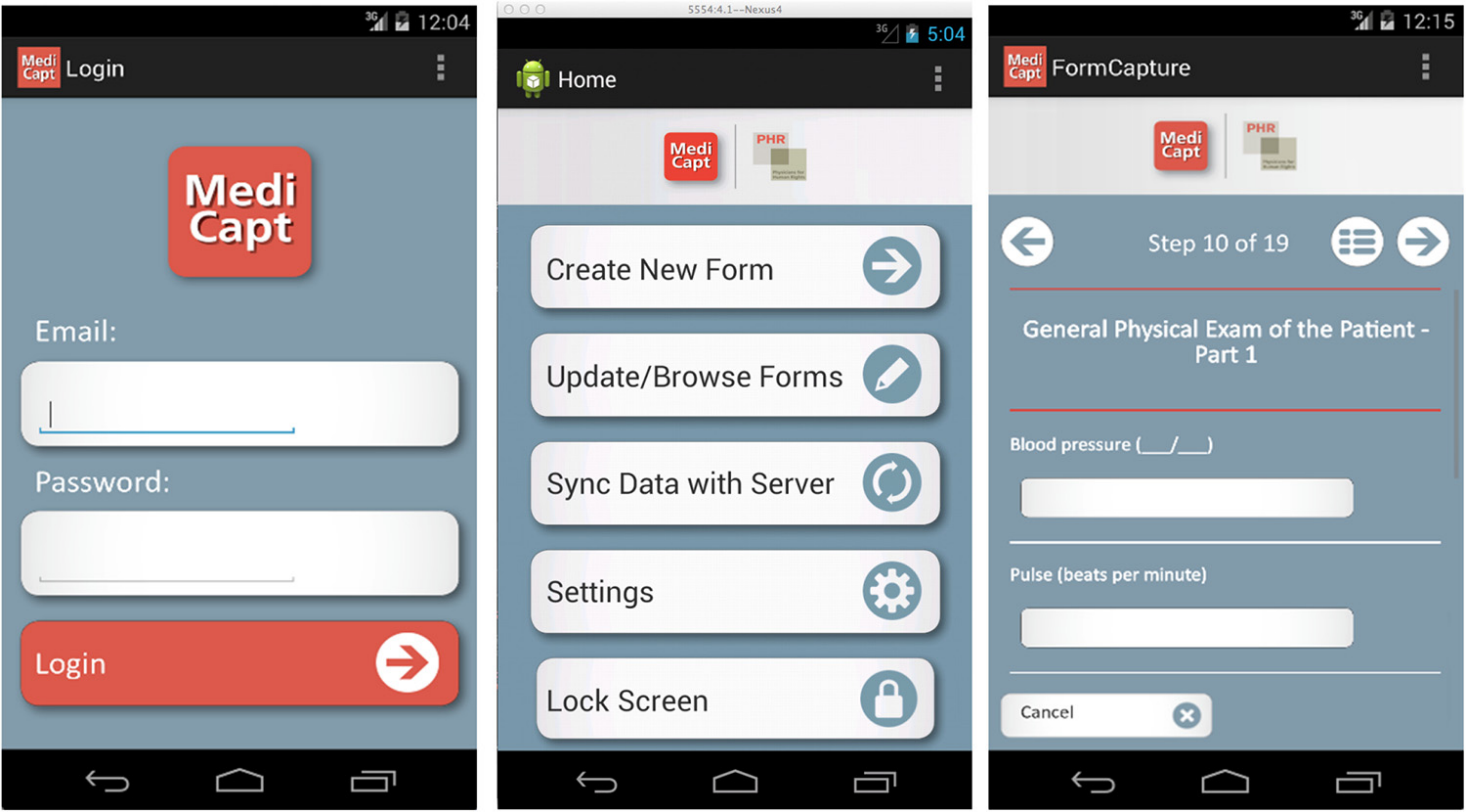
General Architecture of the MediCapt App The screen on the left shows the home screen; the middle screen is the main menu that allows users to complete a new sexual violence form, browse saved forms, and sync their data when connected to the Internet; and the screen on the right shows an example of data-entry fields.

In this article, we describe the early development phases of MediCapt, a first-of-its-kind forensic app, via a participatory design process^16^ with physicians from the DRC who work with survivors of sexual violence, as well as present qualitative findings on their feedback on how to improve the technology and user experience (Figure 1).

## PARTICIPATORY DESIGN METHODS

**FIGURE 2:**
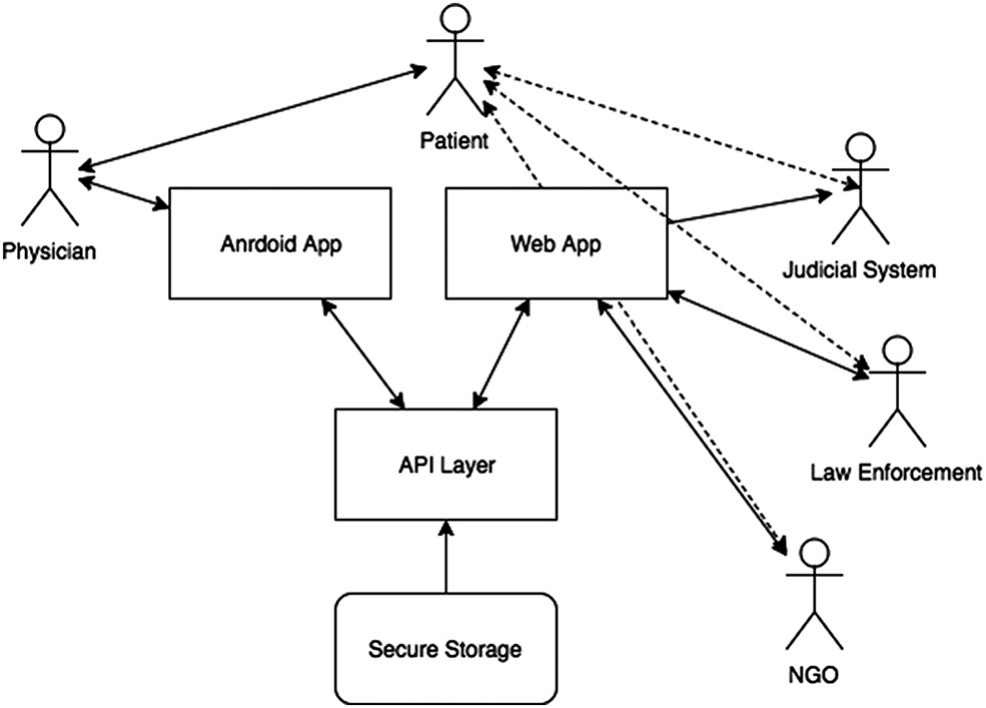
Flow of Sexual Violence Evidence Using the MediCapt App Abbreviation: API, application programming interface.

MediCapt is being designed with 2 goals in mind: (1) to enable health care providers to gather and compile medical evidence related to sexual violence in a standardized manner, and (2) to securely transmit the evidence to authorities engaged in prosecuting and seeking accountability for such crimes (such as investigating officers, gender desk officers in the police force, as well as prosecuting lawyers, magistrates, and judges from the civilian and military justice sectors). MediCapt is meant to circumvent systemic and structural barriers to gathering and sharing evidence related to sexual violence that is particular to low-resourced environments, including conflict zones, by giving health care providers a platform to share that evidence (Figure 2). Although potential end-users of the app cross multiple sectors including the medical, law enforcement, and legal fields, we are focusing initially on meeting the needs of the medical data collectors—the clinicians—and assessing whether the app can be effectively integrated into their workflow.

MediCapt, a smartphone-based app, is intended to enable health care providers to gather medical evidence related to sexual violence and securely transmit that evidence to legal authorities.

As highlighted by several studies^3^^,^^4^^,^^7^ and the Principles for Digital Development,^17^ digital health tool development and implementation benefit from the active engagement of the intended end-users. Development of the MediCapt app to date has included several phases, each involving engagement of intended end-users, including (1) an initial needs assessment, (2) prototype development and field-testing, and (3) prototype refinement and field-testing. Table 1 reviews the MediCapt development process using the Principles for Digital Development as benchmarks, illustrating that our development process closely followed and incorporated many of these benchmarks.

**TABLE 1.** MediCapt Development Process Compared With Principles for Digital Development Benchmarks

Principles for Digital Development^16^	MediCapt Development Process
**Design with the user**	
Develop context-appropriate solutions informed by user needs	✓
Include all user groups in planning, development, implementation, and assessment	✓
Develop projects in incremental and iterative manner	✓
Design solutions that learn from and enhance existing workflows, and plan for organizational adaptation	✓
Ensure solutions are sensitive to, and useful for, the most marginalized populations: women, children, those with disabilities, and those affected by conflict and disaster	✓
**Understand the ecosystem**	
Participate in networks and communities of like-minded practitioners	✓
Align existing technological, legal, and regulatory policies	✓
**Design for scale**	
Design for scale from the start, and assess and mitigate dependencies that might limit ability to scale	✓
Employ a systems approach to design, considering implications of design beyond an immediate project	✓
Be replicable and customizable in other countries and contexts	Planned
Demonstrate impact before scaling a solution	In process
Analyze all technology choices through the lens of national and regional scale	✓
Factor in partnerships from the beginning, and start early negotiations	✓
**Build for sustainability**	
Plan for sustainability from the start, including planning for long-term financial health	In process
Utilize and invest in local communities and developers by default, and help catalyze their growth	Not done
Engage with local governments to ensure integration into national strategy, and identify high-level government advocates	✓
**Be data driven**	
Design projects so that impact can be measured at discrete milestones with a focus on outcomes rather than outputs	In process
Evaluation innovative solutions and areas where there are gaps in data and evidence	✓
Use real-time information to monitor and inform management decisions at all levels	Planned for future
When possible, leverage data as a by-product of user actions and transactions for assessment	Planned for future
**Use open data, open standards, open source, open innovation**	
Adopt and expand existing open standards	Partially done
Open data and functionalities, and expose them in documented APIs	✓
Invest in software as a public good	✓
Develop software to be open source by default with the code made available in public repositories and supported through developer communities	Planned for future
**Reuse and improve**	
Use, modify, and extend existing tools, platforms, and frameworks when possible	✓
Develop in modular ways favoring approaches that are interoperable over those that are monolithic by design	✓
**Address privacy and security**	
Assess and mitigate risks to the security of users and their data	✓
Consider the context and needs for privacy of personally identifiable information when designing solutions and mitigate accordingly	✓
Ensure equity and fairness in co-creation, and protect the best interests of the end-users	✓
**Be collaborative**	
Engage diverse expertise across disciplines and industries at all stages	✓
Work across sector silos to create coordinated and more holistic approaches	In progress
Document work, results, processes, and best practices, and share them widely	✓
Publish materials under a creative commons license by default, with strong rationale if another licensing approach is taken	✓

Abbreviation: API, application programming interface.

### Phase I: Needs Assessment

Between January 2011 and October 2011, we conducted 2 needs assessments in eastern DRC to explore general approaches to the documentation of sexual violence. A multidisciplinary team of U.S.-based clinical, legal, and policy experts conducted key informant interviews with several stakeholders at various sites, including clinicians at 3 major hospitals in Bukavu and Goma; members of local NGOs; lawyers and police officers; and civilian and military justice officials. The interviewees were asked open-ended questions about barriers, gaps, and deficits, each in their respective fields, when encountering cases of sexual violence. The interviews, conducted in French via translators, were summarized and the multisectoral input was used to design PHR's sexual violence program, training workshops, and the paper-based Standard Sexual Violence form.

### Phase II: Prototype Development and Field-Testing

Between October and December 2013 we converted the paper-based Standard Sexual Violence form to digital format (MediCapt 1.0) using simple logic features through the Magpi platform (http://home.magpi.com/).^18^

Magpi—one of the leading public health mobile open-source data collection platforms in the world—was selected initially because it was a third-party platform allowing for both the collection and aggregation (and eventual analysis) of data. It was especially appealing because non-programmers could easily develop customizable forms on the application and the interface was user-friendly.

MediCapt was developed for use on Android phones (and can also run on Android tablets) because of their availability in the DRC, the ease of developing and building Android applications, and the easy-to-use and high-quality cameras on Android phones that allow for the collection and storage of photographs alongside other digital data files.

In January 2014, we invited 8 Congolese physicians who had previously participated in PHR's sexual violence training sessions on medico-legal documentation to participate in a 2-day session to test the MediCapt 1.0 prototype. The session included training on how to use an Android phone, explanation of the Magpi platform, practice using the MediCapt app, and a focus group discussion on the experience, functionality, usability, and feasibility of the app in its early, prototype form. During the focus group discussion, we encouraged clinicians, using open-ended and semistructured questions, to describe what features they deemed important and would like to see in a future version. A questionnaire was administered to participants assessing their background characteristics and general experience with the use of smartphone technology.

Following the 2-day session, physicians were asked to use the prototype app while completing mock patient scenarios and to send feedback to the U.S.-based development team. The collective input was used to design the next prototype, MediCapt 2.0.

### Phase III: Prototype Refinement and Usability Assessment

In January 2015, we invited 9 clinicians who had previously participated in PHR's multisectoral documentation training—3 of whom had used MediCapt 1.0—for a 1-day session with members of the MediCapt development team. The session included a demonstration of MediCapt 2.0, an examination of new and desired features, and in-depth key informant interviews about clinicians' workflow and perceived barriers to the use and integration of this revised tool in the clinical setting. Participants also completed a questionnaire that included questions about background characteristics of the participants and attitudinal questions that were informed by the use of an implementation science framework.^19^ Attitudinal responses were examined using a 4-point Likert scale and captured several domains of implementation including: (1) experience with smartphone-based MediCapt; (2) experience with tablet-based MediCapt; (3) usability of MediCapt; (4) appropriateness of MediCapt; (5) acceptability of MediCapt; and (6) feasibility and sustainability of MediCapt.

**Figure:**
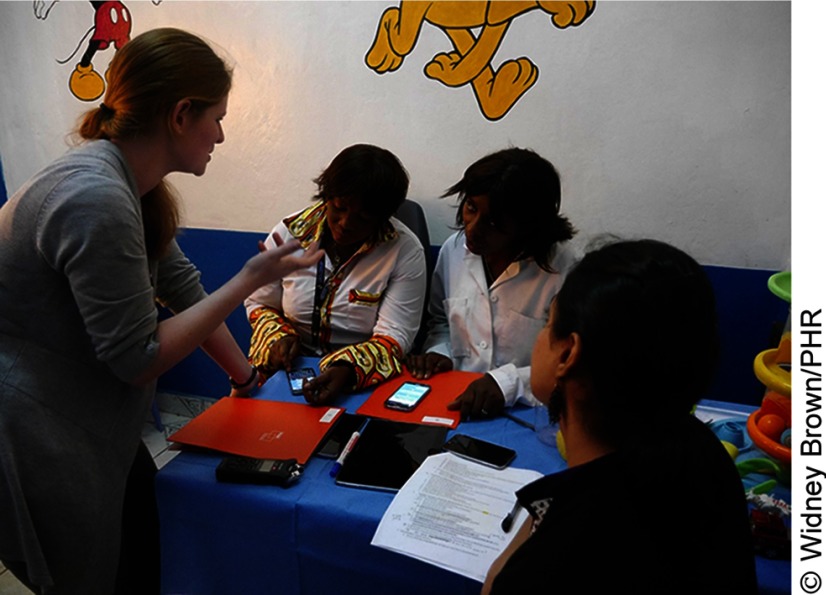
Congolese clinicians at Panzi Hospital in the Democratic Republic of the Congo participate in the collaborative design process of MediCapt.

### Data Analysis

Members of the PHR-MediCapt development team, comprising clinicians, human rights lawyers, and technical experts, conducted the focus group discussions and key informant interviews during phases II and III. Qualitative data were analyzed for emergent themes that could be used to directly improve the technology and user experience of MediCapt. In the Phase III questionnaire, Likert-scale responses from the phase III questionnaire were recoded to numerical values (I strongly disagree=1; I disagree=2; I agree=3; I strongly agree=4) and averaged across participants to create mean scores for each question. We then transformed all Likert scale responses onto a 0–1 scale to ease interpretation, with 0 representing the least level of agreement and 1 representing greatest level of agreement.

Data were entered into Microsoft Excel and analyzed using SAS statistical software. Written consent was obtained from all participants. The questionnaire and protocol were reviewed by the Institutional Review Board (IRB) of RTI International.

## FINDINGS

### Needs Assessment Findings

Interviews with representatives from the medical, legal, and law-enforcement sectors revealed the following themes:

1. Forensic medical exams are rarely conducted or they are conducted poorly.
2. Medical charts fail to document findings sufficiently.
3. Storage and preservation of data are limited or nonexistent.
4. Aggregate data analysis is difficult to impossible to conduct due to gaps in storage.
5. Communication breakdowns between sectors (medical, law enforcement, legal) occur frequently.

Informed by the identified gaps, the objectives of PHR's sexual violence program evolved to address a number of other areas in addition to enhanced collection of evidence, including workforce development, strengthened reporting and dissemination of cases, enhanced communication between various sectors, and improved future surveillance and early detection.

### Feedback on the MediCapt 1.0 Prototype

The focus group participants during the first prototype testing phase (N=8) were clinicians (n=5) or data managers (n=3), and the majority (n=7) practiced in a hospital (the other 2 worked in a mobile clinic). Almost all participants (n=7) were younger than 50 years old, and there was no gender discrepancy (half were men and the other half were women).

When asked about previous use of mobile phones, 6 respondents indicated they had ever used a *regular mobile phone* in the past, while 5 reported having used a *smartphone* (these types of phones were defined on the questionnaire). All participants said they were currently using a mobile phone (6 use only a regular mobile phone and 2 use both a regular mobile phone and smartphone). Half of the health workers indicated they felt very comfortable using both a regular mobile phone and a smartphone, and only 1 worker reported feeling very uncomfortable using them. In addition to making and receiving calls, the other most common reasons that health workers report using their phones were to send and receive texts (n=6 for regular phones; n=4 for smartphones), take photos (n=5 for regular phones; n=4 for smartphones), and connect to the Internet (n=3 for regular phones; n=4 for smartphones).

Participants were also asked to select their level of agreement with several statements related to their confidence in using the MediCapt technology and their beliefs about sexual violence documentation. The majority (n=7) of the respondents felt confident that they could master the use of both a smartphone and a mobile application. All the respondents believed that a mobile application would be useful in their professional work and practice in the future. When asked about documentation of sexual violence evidence, all but 1 health worker believed that documenting evidence of sexual violence using a mobile phone would have a positive impact on bringing sexual violence cases to justice and that the documentation should include an option to take photos of physical findings.

During field-testing of a MediCapt prototype, 7 of 8 users thought such an app would have a positive impact on bringing sexual violence cases to justice.

**FIGURE 3:**
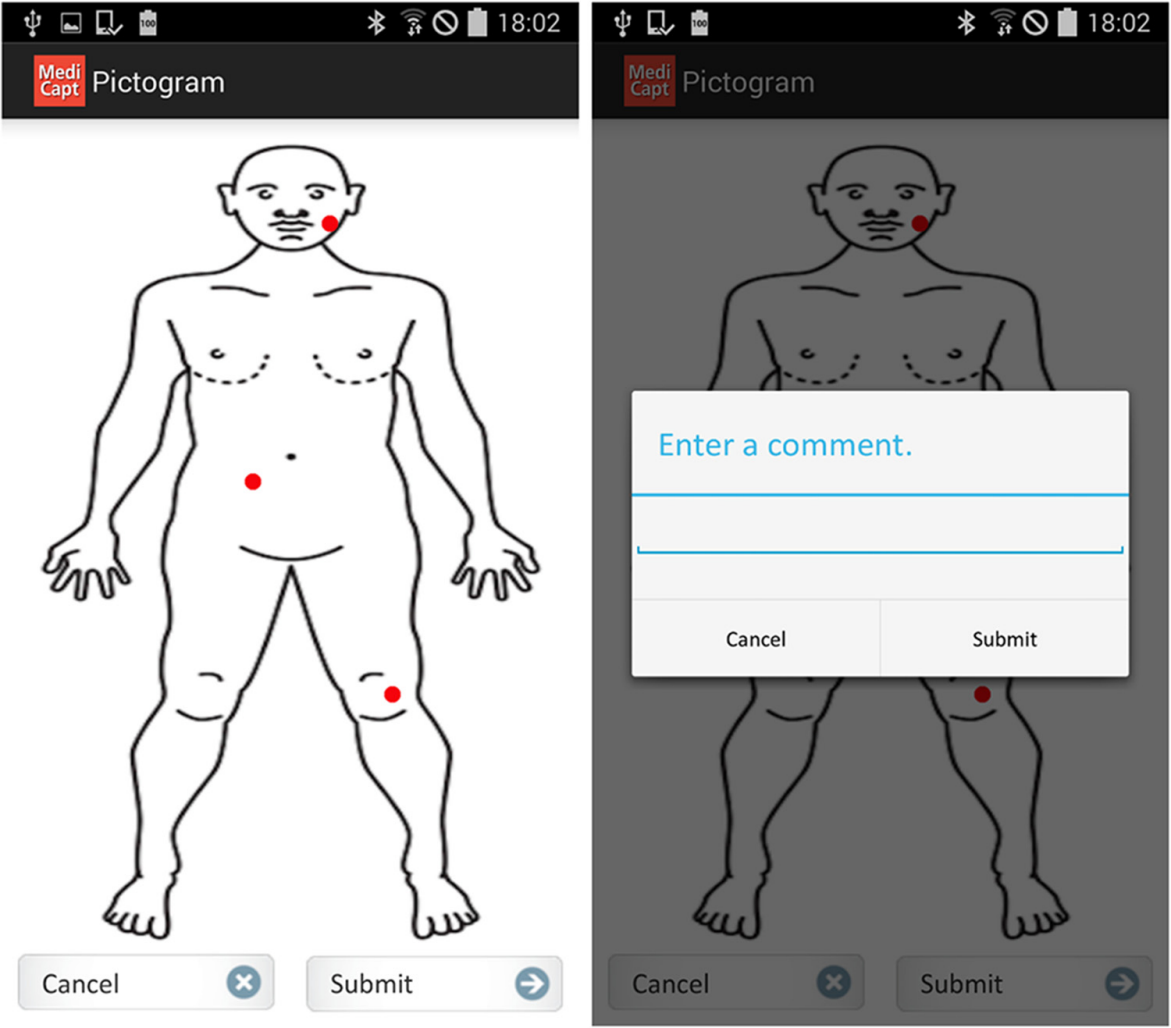
Pictogram Feature in the MediCapt App to Document Location and Type of Injuries Left: The provider can show the location of the patient's injuries on a pictogram, shown with red dots. Right: The provider can also include additional clinical data (e.g., type, size, and depth) about a specific injury.

During the focus group discussion, participants provided feedback about what they thought were important features to include in the MediCapt app based on priority level (i.e., must-haves, should-haves, and could-haves) (Box). Some of the must-have features were foundational features of the first prototype, namely that it provided a digitized version of the paper-based Standard Sexual Violence form and offered secure data encryption. Some other features identified by participants as high priority were incorporated into the next iteration of MediCapt. For example, version 2.0 included the ability to take, store, and transmit photos as part of the evidence package, as well as a pictogram of the body that clinicians could draw on with the touch screen to identify parts of the patient's body affected by the assault (Figure 3). In addition, the participants deemed it necessary to have some type of physical, recognizable manifestation of the data entered into MediCapt—that is, a printed form—in order to document and store the information in health care facilities' print files and also because patients expect to receive a hard copy of their charts. Thus, in version 2.0 we added printing capabilities to allow clinicians to print a form with the completed data if needed.

BOX **User Feedback on Desired Features in the MediCapt App by Priority Level**

Must-Have Features:

- Digitized version of the paper-based Standard Sexual Violence form
- Photo capture capability
- Secure data encryption
- Storage and transmission of data
- Chain of custody preserved
- Geocoding ability
- Printing capability
- Writable pictogram of the body to draw on with touch screen

Should-Have Features:

- Ability to link photo of injury to pictogram image
- Capacity to take photographs as clinician conducts the medical exam
- Ability to annotate photograph
- Option to insert e-signature
- Ability for patient to indicate his/her informed consent

Could-Have Features:

- Scanner feature
- Voice recognition
- Ability to audio record patient's narrative

User testing of the MediCapt protoype informed design of the next iteration including addition of photography, pictogram, and printing features.

Many of the features that users identified as important to include in the next iteration of MediCapt were not possible using the Magpi platform. After conducting a mapping exercise of existing technologies and platforms, we determined there were no off-the-shelf technology platforms that met these articulated needs. Therefore, we decided to develop a unique app from scratch.

### Usability Assessment of MediCapt 2.0

All 9 participants in the Phase II usability assessment were physicians practicing in various locations within the Eastern DRC and had spent at least 3 years conducting sexual assault examinations. The majority (n=5) had previous experience using apps on a smartphone, and some had used a camera on a phone (n=3) or a digital camera (n=4).

More than half (n=5) of the respondents reported taking forensic photographs while conducting sexual assault examinations. Of those who ever used a smartphone (n=5), the most popular app was the camera app (n=3) and WhatsApp (a free messenger service) (n=2).

#### Attitudinal Questionnaire

Overall, respondents had slightly less positive **usability** ratings for using MediCapt on smartphones compared with tablet devices (Supplement Table). However, participants reported greater ease with holding the smartphone than the tablet and greater suitability of the smartphone for documenting sexual assault examinations. Respondents thought it was easy to take photographs with both the smartphone and tablet.

Respondents had favorable responses to items within the **appropriateness** domain (Supplement Table). In particular, respondents had positive opinions concerning the promise of MediCapt in helping personnel do a better job of documenting sexual assault and saving time in conducting sexual assault examinations.

In the **acceptability** domain, respondents indicated (1) they would likely need special training in order to use MediCapt with a patient during an exam, (2) there would be cases where they would not use MediCapt with a sexual violence patient, and (3) existing practices of completing a paper-based medical certificate for examinations of sexual violence patients may make acceptability of technology more difficult (Supplement Table). Still, respondents had generally favorable attitudes about their patients accepting use of MediCapt during examination and feeling comfortable themselves with using MediCapt in clinical practice.

Regarding **feasibility and sustainability,** the largest area for improvement was that additional measures would need to be put into place to make sure the device gets used (Supplement Table). In particular, the respondents perceived it would be difficult to get reliable Wi-Fi or Internet access to transmit the files or even to have electricity to be able to charge the phones daily. In this domain, the most favorable responses were that (1) the respondents could foresee using MediCapt at their health care center, (2) they could train other colleagues on how to use MediCapt, (3) MediCapt would save time in documentation, and (4) MediCapt would ensure records are transferred to the appropriate law enforcement and legal personnel. In addition, the respondents generally felt that MediCapt was intuitive to use—even though their responses in the acceptability domain suggested they needed special training to use the app.

Overall, the highest positive ratings were related to the helpfulness of the app in documenting sexual assault examinations and the ease with which they could take forensic photographs using MediCapt. The lowest ratings were related to smartphone size (too small), tablet size (too large), ease in learning to use MediCapt during an examination, and feasibility of adopting MediCapt for use in practice.

#### Key Informant Interviews

Key informant interviews identified multiple barriers to integrating MediCapt into the workflow in their respective health centers, which fell into 3 groups: infrastructural, systemic and organizational, and personal behavior (Table 2). The main barriers related primarily to contextual elements such as the strong cultural preference for the provision of stamped paper copies. It was noted by all interviewees that various stakeholders (e.g., patients, police) fully expect to have a hard copy of the report; also noted was the existing habit of documenting the findings in multiple places including the medical chart, carnet (small booklet containing a patient's medical information that the patient keeps), and the Standard Sexual Violence form.

**TABLE 2.** Barriers to Integrating MediCapt Sexual Violence Documentation App Into Existing Workflows Reported by Key Informants (N=9)

Type of Barrier	Examples
Infrastructural	Frequent periods with no electricity No Wi-Fi availability during electricity stoppage time Lack of clarity regarding data storage, cloud location, and capacity Limited or no availability of printers and copiers and their associated supplies
Systemic and organizational	Questions regarding organizational support of project (at hospital, district, regional, and national levels) Long-standing workflow practices that promote redundancy and inefficiency (need for multiple copies including the patient chart, carnet, and Standard Sexual Violence form) Need to train multiple clinicians in using app and allowing clinicians time off for training Limited or no availability of electronic medical record system or links to hospital archives
Personal behavior	Educational barriers for technology use (minimal) Personal leadership attributes that affect workflow within health care facility Presence of and ability to negotiate perceived jealously and peer resentment Degree of willingness to try new things Degree of willingness to invest more time initially in learning and using app

**Figure:**
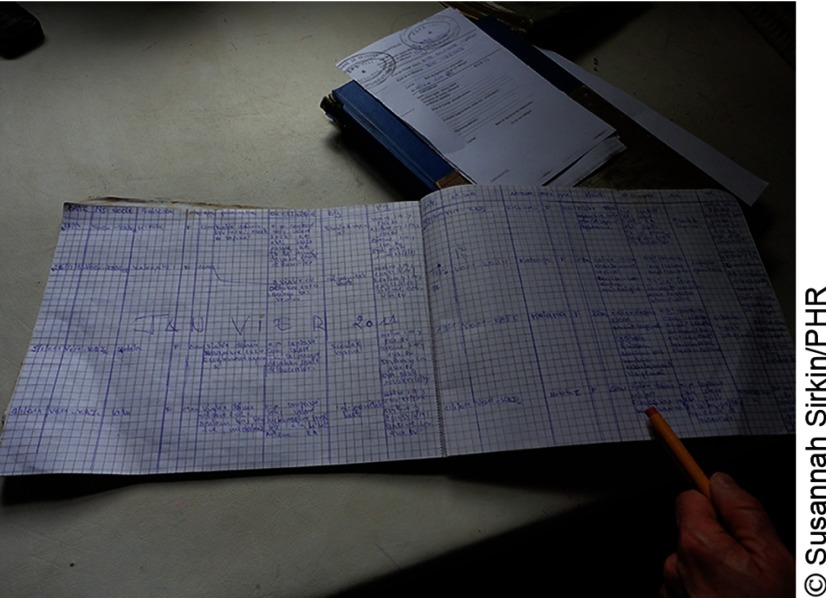
Documentation of sexual violence cases in the Democratic Republic of the Congo commonly entails use of several paper forms, including general ledgers, patient charts, and carnets.

During a second round of field-testing, users suggested the main barriers to integrating MediCapt into their workflows would be shifting cultural preferences for stamped paper copies of sexual violence evidence collection.

## DISCUSSION

We describe a collaborative development and design process with Congolese end-users of a novel human rights app for clinicians intended to standardize the documentation of sexual violence for forensic and legal purposes. NGOs working at the global, regional, state, and local levels are increasingly looking to mobile technology as a tool for the collection of documentation and evidence. To the best of our knowledge, this is the first report of a digital application in the field of human rights and forensic medicine designed specifically to incorporate local legal and forensic needs.

### Current State of Development of the MediCapt App

In its current state, MediCapt is an Android-based app built with a NoSQL, eventually consistent database platform called Couchbase. Eventually consistent means changes made on 1 machine with a copy of the database, such as a user's mobile app, will eventually be transmitted to all other machines with that database, thus allowing the mobile app to work in cases without Internet connectivity through delayed, secure upload of information to the web-based system for use by external parties, such as police and justice officials.

All medical records, photos, and diagrams are stored using 256-bit Advanced Encryption Standard (AES) encryption. To ensure authenticity, metadata, including geolocation, network connectivity, and several other phone or tablet sensor data, are available. All activity is logged, and access is roles-based for security and authenticity. The photography technology relies on built-in hardware intended to bypass regular system storage to protect against access to the underlying data. For security and confidentiality purposes, the photos are stored within the local database on the app and *not* within the photo gallery of the phone.

### Going Paperless

User feedback on MediCapt highlighted the challenges with successfully transitioning workflows from paper-based systems to digital data. In the DRC, one important issue is the lack of electronic health record systems in most health care settings. Clinical encounters, even if captured digitally, still have to be printed and stored in paper forms and in existing paper charts. This creates additional steps for app users, rather than simplifying the process. Moreover, material restrictions (e.g., lack of available printers and ink, copiers and supplies, interruptions in electricity and wireless services) further complicate this transition. The expectation of other sectors and stakeholders (patients, police, administrators) to receive paper copies of sexual violence reports is another issue to overcome in digital data collection and recordkeeping. To address these issues, we have added a Bluetooth-based printing capability, so that clinicians can print the completed forms and give them to the patient at the point-of-care, or any other stakeholders, when requested. At this point in time, we have yet to transfer data to law enforcement or legal authorities. Later phases of MediCapt development will include the transfer of these data to the police, then to local lawyers and judges, but only after specific training of these sectors. Any efforts to implement use of this app in its paperless form will have to include the education and buy-in of multiple players.

Lack of electronic health record systems in the DRC poses an important challenge to transitioning workflows from paper-based systems to digital data.

### Data Security, Privacy, and Safety

Digital health technologies produce digital evidence, which is often difficult to manage and poses issues related to data security.^20^ Data security and privacy were issues that arose early on with participants, owing primarily to the sensitive nature of the information and evidence collected. National or regional regulations with clear legal and policy guidelines are yet to be developed in that setting to ensure medico-legal data are properly protected, transmitted, and stored. Once information is collected by MediCapt in the clinical setting, there remain challenges in ensuring that it is correctly transmitted to the police officer responsible for the case, while adhering to a battery of password protections and other security measures to safeguard the sensitive documentation. This handover is a significant hurdle to consider as we seek to launch the app. We also are considering other unique challenges and pressing questions related to using digital technology for forensic data collection and documentation including how different legal systems permit or exclude electronically stored evidence, what responsibility the developers have to the end-users who may be under great security threats as they use the app to collect forensic evidence, and who ultimately owns the information.

It is important to consider that currently common practice means that sensitive documentation (medical records, court records) are kept in the open, piled up on desks, or strewn on the floor in clinics, hospitals, and court facilities for anyone to access, compromise, or destroy. MediCapt aims to bring some measure of security and protection to ensure those records are stored in a secured, encrypted space, allowing only those key individuals with passwords to access the sensitive materials. There is a risk that third parties may hack into the system where the aggregated and consolidated sensitive materials are stored. However, we have retained a dedicated expert to build out strong security mechanisms to minimize that risk. We are also retaining an independent security expert to run a security audit to expose any potential weaknesses or gaps in the system. And we plan to make the app open-source code to allow for the widest opportunity for independent technologists to test and maintain the code and help us identify and rectify any issues that may arise.

### Partnerships and Operational Capacity

The MediCapt development process has highlighted the importance of building an app within a larger ecosystem of training and partnerships and within an enabling environment. In the participatory context of this endeavor, MediCapt represents 5 years of ongoing conversations and feedback with professionals responsible for the collection of forensic evidence in sexual assault cases. Because the tool itself is a digital version of the documentation codeveloped and tested in-country, the Standard Sexual Violence form itself is known to end-users as credible and acceptable in local courts. Moreover, PHR has worked with key stakeholders in the hospitals and police force, as well as with justice officials at the grassroots level and government and ministry leaders at the highest levels. This has helped PHR and the MediCapt development team obtain the national political investment necessary to be able to pilot the app.

The participatory context of MediCapt's development and its alignment with local legal and regulatory policies places a premium on long-term, strategic, political, and technical investment in the stakeholders and the community where the app will be used. The challenge, of course, is the organization's ability to do that in other countries and settings and raises the question of scaling up and disseminating the app in a meaningful way elsewhere.

Another challenge is the need to invest heavily in technical human resources not only in the design phase but also at launch to make sure there is a dedicated IT person on the ground for troubleshooting at each point of use (at the hospital, police station, or court). This may be particularly burdensome for NGOs that are not traditionally used to handling and managing long-term technology projects.

Such organizations are advised to work closely with partners who have technical, political, and financial expertise from the earliest stages of the initiative, even before embarking on technological design. The use of validated toolkits will help anticipate and mitigate common hurdles experienced by many in the mHealth field. A particularly useful one is the mHealth Assessment and Planning for Scale (MAPS) toolkit—a self-assessment tool that guides project teams when developing or scaling up their innovations.^21^

### Building for Sustainability

We have developed and built out MediCapt so that it can be scalable. This article describes early phases of development of the app in the DRC. However, we are working on extending the MediCapt pilot to Kenya in the near future, and comparing the experiences of users in these 2 very different settings. MediCapt has been designed to be ultimately used in many countries and it has been future-proofed to allow custom configuration of any standard national forms documenting not only sexual violence but also other kinds of human rights violations including torture, as well as configuration in multiple languages.

### Limitations

Our study has several weaknesses. First, our project was carried out with a small number of participants in a unique setting with its own cultural and structural barriers. While this approach is appropriate for the specific purpose of obtaining qualitative feedback from intended end-users on prototypes and we believe our conclusions to be useful in informing future iterations of the app, the findings cannot be generalizable to other locales, sectors, and clinical data collection purposes. Second, during this pilot phase, we did not compare data collection with the app with the current standard process of collecting data with paper forms to assess accuracy, completeness, and ease of use, among other possible outcomes. We plan to evaluate these issues in the near future, via a "standard practice" control group. Finally, our participants' input and experience may not reflect those of others in their field or geographic location because of inherent differences in education, technological savviness, and technical expertise.

## CONCLUSION

Despite the increased usage of digital health applications, the majority have not been tested and properly evaluated.^22^^–^^24^ Our article describes the preliminary user feedback on a mobile app in development, MediCapt, to document sexual violence evidence for forensic and legal purposes. In order to assess the efficacy and effectiveness of the app, a robust monitoring and evaluation process has been developed and will be carried out longitudinally. Determining the true impact of MediCapt on medical, legal, and human rights outcomes will require years of study and certain methodological revisions.

It is critical that the human rights and technology communities come together to explore these issues that cross a wide range of areas—not only technological but also legal and ethical. Future consideration and development of operational, technical, and legal frameworks may help NGOs ensure that the collection of evidence and documentation of human rights abuses using digital technology is done in a manner that enhances local capacity, strengthens documentation, reduces risks to the documenter, survivor, and witness, and promotes human rights principles and justice.

It is critical for the human rights and technology communities to explore together the technological, legal, and ethical issues posed by use of digital technology to document human rights abuses.

Finally, whereas digital health, and specifically mHealth, focuses on *data* collection for medical, health, and public health purposes, we believe that our experience of collecting data that will potentially serve as *evidence* broadens the traditional scope of digital health and mHealth. As more human rights-oriented NGOs rely on mobile technology for digital evidence collection, we see a need to refine and define this as a unique field of exploration, which we would like to term mobile justice, or mJustice.

## Supplementary Material

Supplemental material

## Notes

**Peer Reviewed**

**Competing Interests:** Ranit Mishori is a paid medical consultant for Physicians for Human Rights' Program on Sexual Violence in Conflict Zones and the MediCapt project. Hope Ferdowsian is a paid medical consultant for Physicians for Human Rights' Program on Sexual Violence in Conflict Zones. Michael Anastario is a paid consultant for Physicians for Human Rights' Program on Sexual Violence in Conflict Zones. Karen Naimer is Program Director, Physicians for Human Rights' Program on Sexual Violence in Conflict Zones. Sucharita Varanasi is the former MediCapt Project Manager and Senior Program Officer, Physicians for Human Rights. Dori Able has no competing interests. Kevin Chugh is a paid technology consultant for the MedicCapt project.
