# Supplementary material for: mJustice: Preliminary Development of a Mobile App for Medical-Forensic Documentation of Sexual Violence in Low-Resource Environments and Conflict Zones
Source: Glob Health Sci Pract. 2017 Mar 24;5(1):138–51. doi: 10.9745/GHSP-D-16-00233 (PMC5478223; doi:10.9745/GHSP-D-16-00233)
Supplement: Supplemental material [file supp_5_1_138__index.html]

Supplemental material 

# mJustice: Preliminary Development of a Mobile App for Medical-Forensic Documentation of Sexual Violence in Low-Resource Environments and Conflict Zones

## Supplemental material

- Text s01, PDF - Text s01, PDF
