# Supplementary material for: mJustice: Preliminary Development of a Mobile App for Medical-Forensic Documentation of Sexual Violence in Low-Resource Environments and Conflict Zones
Source: Glob Health Sci Pract. 2017 Mar 24;5(1):138–51. doi: 10.9745/GHSP-D-16-00233 (PMC5478223; doi:10.9745/GHSP-D-16-00233)
Supplement: Supplemental material [file GHSP-D-16-00233_16-00233-Mishori-Supplement.pdf]

Mishori R, Anastario M, Naimer K, et al. mJustice: preliminary development of a mobile app for medical-forensic documentation of sexual violence in low-resource environments and conflict zones. Glob Health Sci Pract. 2017;5(1). http://dx.doi.org/10.9745/GHSP-D-16-00233

SUPPLEMENT TABLE. Average Usability Scores for the MediCapt 2.0 Prototype App (N=9)

	Mean	SD
**Usability on Mobile Phones**		
The screen of the smartphone is too small.	0.41	0.22
The smartphone should be larger.	0.67	0.29
It is easy to type on the smartphone.	0.82	0.17
It is easy to use the touch screen on the smartphone.	0.82	0.24
The smartphone itself appears to be suitable to document sexual assault examinations.	0.85	0.17
It is easy for me to hold the smartphone.	0.85	0.17
It is easy to take photographs using the smartphone.	0.93	0.15
**Usability on Tablets**		
The tablet should be smaller.	0.41	0.36
The screen of the tablet is too large.	0.55	0.37
The tablet itself appears to be suitable to document sexual assault examinations.	0.78	0.24
It is easy for me to hold the tablet.	0.78	0.29
It is easy to type on the tablet.	0.89	0.17
It is easy to use the touch screen on the tablet.	0.89	0.17
It is easy to take photographs using the tablet.	0.93	0.15
**Appropriateness of the MediCapt App**		
It will be easy to use MediCapt while I am conducting a sexual assault examination on a patient.	0.89	0.17
My patients will be better served if I use MediCapt.	0.89	0.17
MediCapt will help me save time in conducting sexual assault examinations.	0.93	0.15
MediCapt will help me do a better job of documenting sexual assault examinations.	0.96	0.11
**Acceptability of the MediCapt App**		
The use of MediCapt with a sexual violence patient is culturally unacceptable.	0.19	0.17
There are cases where I would NOT use MediCapt with a sexual violence patient.	0.41	0.33
I would need special training in order to use MediCapt with a patient during an examination.	0.52	0.34
I currently complete a paper-based medical certificate for examinations of sexual violence patients.	0.63	0.26
I think that the forensic photography function on MediCapt will make it more comfortable for my patients to be photographed, versus using a separate camera to take photographs.	0.78	0.17
I think that sexual violence patients would accept my use of MediCapt during their examination.	0.82	0.17

Mishori R, Anastario M, Naimer K, et al. mJustice: preliminary development of a mobile app for medical-forensic documentation of sexual violence in low-resource environments and conflict zones. Glob Health Sci Pract. 2016;5(1). http://dx.doi.org/10.9745/GHSP-D-16-00233

	Mean	SD
I would be comfortable using MediCapt in my clinical practice.	0.89	0.17
I like to use new types of technology to help my patients.	0.93	0.15
Overall, I am satisfied with MediCapt.	0.93	0.15
**Feasibility and Sustainability of the MediCapt App**		
It will be difficult to charge the smartphones or tablets on a daily basis.	0.26	0.32
The device is likely to get stolen.	0.29	0.11
I may have to rely on a generator to charge smartphones or tablets on a daily basis.	0.30	0.35
I am likely to lose my device.	0.33	0.17
I think connection to the Internet will be a major problem for uploading files.	0.37	0.35
I expect that it will be difficult to get reliable Wi-Fi or Internet access to transmit the files.	0.48	0.41
Additional measures will need to be put into place to make sure this device gets used.	0.59	0.15
MediCapt is better than what I am currently using to document sexual assaults.	0.79	0.25
The app “made sense.”	0.82	0.17
I will be able to take forensic photographs easily using MediCapt.	0.82	0.34
I have Wi-Fi or other Internet access in my community health care center.	0.82	0.17
I have access to reliable electricity in my community or health care center.	0.85	0.17
My colleagues will be happy using MediCapt.	0.89	0.17
I have had enough training to use MediCapt correctly.	0.89	0.17
Health care professionals who use MediCapt will take better forensic photographs because they are using MediCapt.	0.89	0.17
MediCapt is intuitive to my needs when documenting sexual assault examinations.	0.93	0.15
In reality, I could see MediCapt being used at my health care center/clinic.	0.93	0.15
I could one day train my colleagues on how to use MediCapt to document sexual assault examinations.	0.93	0.15
MediCapt will help me save time in documentation.	0.93	0.15
MediCapt will ensure that sexual assault records are transferred to the appropriate law enforcement and legal personnel.	0.93	0.15

Abbreviation: SD, standard deviation.
